# The Effect of Telenursing on Disease Outcomes in People with Type 2 Diabetes Mellitus: A Narrative Review

**DOI:** 10.1155/2023/4729430

**Published:** 2023-12-07

**Authors:** Mina AkbariRad, Mohsen Dehghani, Masoumeh Sadeghi, Ashkan Torshizian, Nikoo Saeedi, Mehrdad Sarabi, Mahdieh Sahebi, Mohammad Taghi Shakeri

**Affiliations:** ^1^Department of Internal Medicine, Faculty of Medicine, Mashhad University of Medical Sciences, Mashhad, Iran; ^2^Department of Epidemiology, Faculty of Health, Mashhad University of Medical Sciences, Mashhad, Iran; ^3^Metabolic Syndrome Research Center, Mashhad University of Medical Sciences, Mashhad, Iran; ^4^Islamic Azad University, Mashhad Branch, Mashhad, Iran; ^5^Department of Biostatistics, Faculty of Health, Mashhad University of Medical Sciences, Mashhad, Iran

## Abstract

**Method:**

A comprehensive search of online databases, including PubMed, Scopus, Cochrane Library, and Google Scholar, was performed using the following MeSH keywords: telenursing, telephone follow-up, diabetes mellitus, disease management, glycemic, self-care, treatment adherence, and quality of life, up to September 2023. Two reviewers independently screened pertinent studies based on the prespecified outcomes (treatment adherence, self-care, glycemic control, and quality of life) and extracted data from all eligible studies.

**Results:**

Of all retrieved records, 23 studies including 5 quasiexperimental (21%) and 18 randomized controlled trials (RCTs) (79%) from five continents met the inclusion criteria. Both male and female patients were considered in the included studies, with mean age of 56.2 years old and a follow-up range of 12 weeks to 18 months. Findings showed that telenursing or nurse telephone follow-up significantly increased mean self-care efficacy score, improved adherence to the treatment regimen, decreased glycosylated hemoglobin and plasma glucose levels (but not lipid profile and body mass index), and improved quality of life compared to the routine care in people with T2DM.

**Conclusion:**

Telenursing can effectively supplement healthcare professionals to manage PWT2D. Increasing patients' knowledge about their drugs, insulin administration, and diabetes complications improves self-care behaviors and medical adherence. Consistently, improved self-care and regular use of treatment result in improved metabolic indicators and decreased rate of complications, which is associated with a better quality of life.

## 1. Introduction

Diabetes mellitus is the most common chronic disease in the world. As the prevalence of diabetes mellitus is dramatically increasing worldwide, the World Health Organization has mentioned it as a silent epidemic [1]. According to the statistics published by the World Health Organization, 190 million people have diabetes. By 2025, this number will reach more than 330 million people, of which 77.6% are in developing countries. More than 6 million of them are in Iran [2].

Diabetes mellitus causes many complications, such as retinopathy, nephropathy, diabetic foot, depression, chronic kidney disease, and cardiovascular diseases [3]. It is one of the most important causes of morbidity and mortality. Every year, 4 million people die from diabetes complications, and it is estimated that one person dies every 10 seconds [4].

There is no definite cure for diabetes, but it can be controlled. Researchers divide diabetes control into five components: nutrition, exercise, blood sugar control, medical treatment, and patient education [5]. Due to the chronic nature of this disease, continuous management and follow-up of people with type 2 diabetes are of the essence [6]. There are different methods of follow-up care, either in the form of a client's visit to the service provider's centers or being visited at home by the service provider, which has many limitations. These methods require a lot of time, energy, and money [7].

Furthermore, most people with type 2 diabetes are very busy and cannot visit healthcare professionals regularly, or in many cases, these people live in remote areas with limited or no access to healthcare providers [8]. Moreover, service providers are dissatisfied with spending a lot of time providing continuous service to people suffering from chronic diseases such as diabetes [9]. Regarding the mentioned limitations, we require new approaches using new communication methods to follow up with people with type 2 diabetes [10].

One of these methods is telenursing, which uses information technology and remote communication in nursing to help physicians and nurses check whether people with type 2 diabetes have enough adherences to the treatment, analyze their condition, and suggest nursing recommendations. This method can potentially increase the ability of people with type 2 diabetes to achieve better control and improve their health status [11]. In general, telenursing is a subset of telehealth and is often used when the distance between the patient and the health center is large [12]. Telenursing uses different tools such as video, Internet, and phone calls. Of these communication devices used in this care method, the phone call is the most available method, which is used more often [13]. By removing the barriers of time and space, telehealth techniques have improved patient relations with their healthcare providers and reduced related costs [14]. In recent years, several studies have been conducted on different telenursing tools used in self-care, diet therapy, paraclinical indicators, and quality of life of people with T2DM [15, 16].

Existing data have reported preliminary evidence on the efficacy of telemonitoring and telemedicine for diabetes care. Telephone consultation with a physician is a frequent healthcare setting in which physician-patient communication skills are exercised [17–20]. Moreover, nurses are among the most effective healthcare staff in telephone consultations. Many randomized controlled trials have investigated whether telenursing positively affects disease management [21–23]. However, the evidence for its effectiveness is limited, and the results are inconsistent [16]. Hence, we conducted a narrative review to further investigate telenursing's role in managing diabetes. We also aim to examine and compare the efficacy rate of telenursing in different areas of diabetes care. We assessed the impact of telenursing on four important outcome domains associated with T2D treatment, explained the results of previous studies, compared them with each other, and discussed the possible causes of their discrepancies.

## 2. Methods

A comprehensive search of online databases, including PubMed, Scopus, Cochrane Library, and Google Scholar, was performed up to September 2023 to identify English language studies. The following MeSH keywords were used for search in databases: telenursing, telephone follow-up, Diabetes, diabetes mellitus, disease management, glycemic control, glycosylated hemoglobin and plasma glucose, self-care, treatment adherence, and quality of life. Two reviewers independently screened the studies based on titles and abstracts, and finally, if required, the full texts were reviewed. The reference list of retrieved studies was also checked for additional pertinent studies. Prespecified outcomes were treatment adherence and diabetic diet, self-care, glycemic control, and quality of life.

### 2.1. Eligibility Criteria

Studies were included in the narrative review if they assessed the effect of telenursing or nurse telephone follow-up in the clinic, healthcare centers, or hospital setting on the glycemic control, lipid profile, self-care, adherence to the diabetes treatment regimen, and quality of life compared to routine care in people with T2DM. Adult human population and peer-reviewed and English-language publications were included. When there were multiple publications from the same population or patients, the most recent report with the most follow-up time was included. Due to the nature of the research question, interventional studies including experiment, quasiexperimental, and randomized controlled trials were included in our review. In this regard, all observational studies (cross-sectional, case-control, or cohort studies), case reports, case series, letters, and short communications were excluded. Interventional studies with less than 12 weeks of follow-up and all diabetic patients other than T2DM were excluded from the review.

Extracted information and characteristics of all studies include the author's last name, year and country of the study population, age, gender, study design, study population and setting, follow-up time, type of intervention (telenursing or nurse telephone follow-up), and study outcomes. Two reviewers independently extracted data from all studies, and differences or discrepancies between investigators were resolved by discussion.

## 3. Results and Discussion

Tables 1–4 show the main characteristics of all included studies in the present narrative review. A total of 4423 records were obtained in the primary comprehensive search. Five hundred and sixty-eight (568) records remained after removing duplicates and studies that were unrelated to the research question. After excluding the studies that did not meet the eligibility criteria (mentioned in the selection criteria), 23 interventional studies fulfilled our inclusion criteria, which included 5 quasiexperimental (21%) and 18 randomized controlled trials (RCT) (79%). Both male and female patients were considered in the included studies, with a mean age of 56.2 years old. The range of follow-up in the included studies was 12 weeks to 18 months. All studies investigated nurse telephone call/follow-up only or plus routine care compared with usual care in people with T2DM and were conducted in the USA, Belgium, Denmark, Turkey, Australia, Thailand, Iran, India, Ghana, Egypt, and China (Tables 1–4).

### 3.1. Self-Care

The knowledge, skills, and abilities required by people with T2DM to help them make informed decisions to achieve better blood sugar control occur through self-management education programs. The self-management education program accounts for the main foundations of diabetes mellitus care. Several studies have shown that self-management education can increase knowledge, improve self-care behaviors, reduce HbA1c, and improve the quality of life in people with type 2 diabetes [24]. Diabetes mellitus is considered a self-control disease, where 11% of its treatment depends on self-care behaviors, and it is possible to reduce 41-85% of its complications by promoting preventive behavior, patient education, and self-care intervention [25]. Researchers consider the lack of self-care interventions as one of the most critical factors behind the death of people with type 2 diabetes [26].

Self-care in patients with type 2 diabetes (PWT2D) includes a wide range of activities, such as regular blood sugar measurement, diet regulation, exercise, timely use of medicines, and foot checks, which require some fundamental lifestyle changes. Continuous self-care intervention is associated with a lower glycosylated hemoglobin (HbA1c) level and fewer complications. Since diabetes causes behavioral and emotional changes, daily control is not an easy task for PWT2D to do. PWT2D tend to neglect day-to-day control because of various preoccupations; therefore, they need continuous follow-ups done by physicians and nurses [27]. Considering the high and growing prevalence and chronic nature of this disease, the follow-up method should apply to a large number of PWT2D [28].

By providing care using communication devices such as telephone, Internet, and video, telenursing will fade the barriers, reduce the costs, and provide access to improved self-care interventions for diabetic patients more effectively. Various studies have confirmed the positive effects of telenursing on the self-care efficacy of PWT2D (Table 1). In 2010, Nesari et al. assessed the impact of telenursing on a variety of diabetic patients' self-care aspects, including adherence to diet, exercise, and foot care, through a randomized trial and observed a notable increase in all domains in patients receiving telenursing compared to patients receiving usual care [29]. Others have performed similar studies; Asante et al. conducted an RCT on PWT2D in Ghana [30]. Elgaphar and El Gafar and Soliman investigated the effect of telenursing on the self-care efficacy of women with type 2 diabetes in Egypt [31, 32]; Azhdari Mamaghani et al. conducted a randomized trial on 156 PWT2D and assessed patient's self-efficacy upon receiving telenursing in Iran [33], and Aytekin Kanadli et al. conducted an RCT in turkey assessing self-care of PWT2D with and without telenursing [34]. The results of all mentioned studies indicate that the self-care efficacy significantly increased after the intervention compared to the control group [30–34]. Shahabi et al. have also recently assessed the impact of telenursing on patients' adherence to diet and concluded that telenursing can successfully change patients' eating habits compared to the usual care [35]. Wong et al. also investigated telenursing's effect on self-care in managing type 2 diabetes. They showed that telenursing significantly increased patients' self-care efficacy regarding exercise adherence. At the same time, no significant difference was observed regarding diet adherence after three months [23]. In contrast, assessing 473 patients in an RCT setting, Blackberry et al. did not detect any changes in the self-care efficacy of PWT2D in a comparison between telenursing and the usual care [36].

Due to lack of motivation, insight, and financial support, a large proportion of PWT2D do not receive diabetes-related special care and adequate outpatient visits [37]. During the last decades, telecommunications and technology have played a vital role in managing PWT2D. Telenursing intervention is not a treatment, and it is aimed at changing the behavior of PWT2D through education. Previous telenursing studies showed that the nurses educated patients about the administration of insulin, observed symptoms of diabetes complications, and improved appropriate use of medical care by different methods of telecommunications technologies [11, 12, 38]. The information that PWT2D receive during telenursing programs enables them to actively participate in their treatment and take responsibility for their health. A large number of studies evaluating self-care have measured different outcomes as self-care. Furthermore, their follow-up durations and the telecommunication methods were different, making their results incomparable.

### 3.2. Adherence to the Treatment Regimen

Adherence to the treatment is defined as the degree of the patient's behavioral compliance with health or treatment recommendations, regular use of medications, modified lifestyle principles, and commitment to the physician's advice. Treatment adherence is a complex behavioral process that depends on several factors, such as the individual characteristics of PWT2D and the mutual relationship between the physician, the patient, and the healthcare system. According to the definition presented by the World Health Organization, compliance is an action by which a person performs a behavior, such as taking medicine, following a diet, or implementing a lifestyle change, following the recommendations provided by the healthcare system [39].

The degree of adherence of PWT2D to treatment regimens directly affects the treatment results, which can lead to their improvement or disability. Unfortunately, in most cases, the patient's adherence to these diets is relatively poor [40]. Even though the complications of diabetes can be prevented or postponed, several studies have reported that the global status of diabetes control is undesirable [41, 42]. Poor adherence to the treatment is one of the main concerns and clinical problems that health system employees have to face many times daily [43]. Nowadays, with the vast advancement of knowledge and technology, especially in communication and information technology, various approaches are available to strengthen treatment compliance, leading to new methods such as telenursing [44]. Telenursing, considered a subbranch to telehealth, is currently offering improvements to healthcare on a global scale. This leads to providing related health requirements; coordinating, managing, and providing care services through communication and information technology; and eliminating cultural, social, temporal, and geographical barriers [45].

Various studies have investigated the effects of telenursing on adherence to the treatment regimen (Table 2). A study conducted by Asante et al. on 60 PWT2D for three months showed a significant improvement in compliance to the treatment regimen in the intervention group compared to the control group [30]. Crowley et al. and Kaur et al. also observed an increased treatment adherence in the telenursing group compared to the usual care [46, 47]. In accordance with this, Bingham et al. performed a systematic review of 13 studies and reported that telenursing was associated with improved outcomes on medication adherence in 8 studies [28]. However, a study by Gatwood et al. examined the impact of tailored text messages on health beliefs and medication adherence of patients with uncontrolled diabetes. After three months, they found no significant difference between the intervention and the control group [48]. Wong et al. also found that there were no significant differences in treatment adherence between telenursing and routine care [23]. These discrepancies can be explained by factors such as follow-up duration, sample size, baseline adherence level, and type of medication.

Evidence from the literature demonstrates that telenursing is associated with significantly improved medical adherence through different routes, such as flashcards and online videos (Table 2). In summary, telenursing impacts medical adherence through one of the following routes: (1) interventions that mainly focus on patient engagement, such as conversation with patients to identify and resolve barriers to medication nonadherence; (2) organizing a plan to help resolve their medication adherence problems; (3) setting alerts to remind patients to take their medications; (4) use of education tools to improve a patient's knowledge with the aim of better understanding the importance of their medications [49, 50]. There are several limitations to the interpretation of previous studies' results, such as the lack of prospective evaluations, absence of controlled cohort studies, unavailability of baseline medication adherence rates, limitations in statistical analysis, and small sample sizes.

### 3.3. Improvement of the Metabolic Indicators

In various studies, the effects of telenursing on different metabolic indicators of diabetes have been investigated (Table 3). For instance, many studies explored telenursing's effect on glycosylated hemoglobin (HbA1c) levels. The impact of telenursing on metabolic indices is quite variable in different studies [33, 51–53]. For example, in a large number of studies (Table 3), supplementation of telenursing has significantly reduced HbA1c levels more than usual care alone in a 12-week period [31, 33, 34, 51, 52, 54–57]. Plausible effects of telenursing have also been observed on the reduction of postprandial glucose (PPG) [31]. In 2020, Asante et al. conducted a randomized clinical trial on 60 African PWT2DM and concluded that telenursing is associated with a more significant decrease in HbA1c compared to the usual care [30]. In line with the mentioned studies, Hansen et al. and Odnoletkova et al. followed 166 and 574 PWT2D, respectively, and observed a significant drop in HbA1c in patients receiving telenursing in addition to usual care compared to ones receiving usual care alone [58, 59]. A recent meta-analysis of 17 clinical trials has also demonstrated that telenursing is associated with a statistically significant reduction in HbA1c and FBS levels [16]. Inconsistent with these findings, Lashkari et al. performed a telenursing strategy on 50 PWT2D and found that the mean reduction of FBS level was not different between the two groups [52]. Similarly, Wong et al. found no significant difference in HbA1c and BMI changes between patients receiving routine care and the ones who received extra-telenursing after 24 weeks [23]. Crowley et al. also conducted an RCT with a relatively large sample size (359 PWT2D) with a one-year follow-up. They observed no significant differences regarding changes in HbA1c between telenursing and usual care groups, even though the telenursing group was more likely to adhere to their treatment regimen [46]. Additionally, Blackberry et al. followed 473 patients for 18 months and concluded that adding telenursing to usual care is ineffective in lowering HbA1c [36].

Besides glycemic indices, the effects of telenursing on other factors, such as lipid profile, have also been examined. A meta-analysis by Yang et al. reported no significant impact of telenursing on total cholesterol levels [16]. The number of studies focusing on the effects of telenursing on BMI of PWT2D is few, and there is no consistency among their results. Telenursing does not show a positive impact on BMI [52]. In Hansen et al.'s study, 165 PWT2D were subjected to a 32-week intervention in a one-time video conference session followed by a 6-month follow-up via computer-based consultation and follow-up. The results showed that telenursing does not significantly affect BMI [58]. In addition, Lashkari et al. showed that receiving weekly telephone calls for 12 weeks did not considerably impact BMI [52]. Stone et al. did not detect any significant difference regarding weight reduction between usual care and telenursing either [57]. On the other hand, Shahsavari and Bakhshandeh Bavarsad conducted an RCT on 60 PWT2D with 12 weeks follow-up. They observed a significant decrease in BMI in the telenursing intervention group, while no changes were seen in the usual care group [56]. It should be mentioned that weight reduction takes more time, education, and care and may not always be observed in short-term follow-ups. Larger sample RCTs with extended follow-up periods are required to make precise judgments on the effects of telenursing on BMI and weight change.

Most of the reviewed studies indicate that telemonitoring interventions and a combination of telemonitoring and teleeducation methods significantly reduced HbA1c in PWT2D [21–23, 34, 46]. In addition, using behavior change and clinical treatment models, either alone or combined, significantly improved metabolic indicators levels [60]. Clinical treatment intervention models notably led to the most significant decline in HbA1c [16]. Telenursing in diabetes includes several aspects, including telemonitoring and teleeducation. The considerable effect of telenursing on diabetes control results from healthy behavioral changes as well as receiving medical treatment. It is worth mentioning that although we found a significant reduction of metabolic indexes throughout the reviewed studies, one of the limitations of this narrative study is the unavailability of patients' baseline levels. Therefore, the efficacy of different telenursing strategies cannot be compared.

### 3.4. Quality of Life (QOL)

According to the definition presented by the World Health Organization, QOL refers to a general state of physical, mental, and social well-being. This definition is a broad concept that is affected in a complex way by physical health, psychological status, level of independence and social relationships, and the specific characteristics of the environment [61]. The term quality of life, specifically health related to the QOL, refers to physical, psychological, and social health, which is influenced by a person's experiences, attitudes, expectations, and perceptions [62].

During the past decades, the changes in the pattern of diseases and the increase in chronic diseases have led to increased attention to the QOL of PWT2D [63]. Diabetes is a systemic disease involving different systems, resulting in psychological and behavioral problems. PWT2D experience changes in their diet, permanent dependence on medicine, numerous short- or long-term complications of the disease, and related costs. These accumulations suggest that diabetes affects the QOL of people. A significant number of studies have reported low QOL in diabetic patients [64–66]. In line with this, the rate of depression in PWT2D is about two times higher than in the normal population [63]. The QOL of PWT2D is of great importance, and one of the major goals of PWT2D is to prevent any deterioration in the QOL [67].

Telenursing is a type of application of information technology in the care of PWT2D, which is used for self-evaluation, monitoring, decision-making, and giving necessary recommendations and is planned based on the care needs of diabetic people when they are not available. In this method, based on age, gender, and health problems, PWT2D share their physical performance status with the healthcare provider and receive the necessary care by using communication devices. It is a very useful and cost-effective method to assess the needs of PWT2D outside of care time and reduces the number of frequent visits [68].

Various studies have investigated the effects of telenursing on quality of life, so far (Table 4). In a study conducted by Magbool et al. on PWT2D, telenursing was an effective nursing strategy in improving the lifestyle and clinical condition of diabetic people. They also recommended that telenursing should be planned as a part of the health plan for diabetic people in different health environments [3]. In another study conducted by Asma et al. on women with gestational diabetes, they presented telenursing as a practical and effective way to improve their QOL [69]. Elgaphar and El Gafar conducted telenursing intervention on 50 PWT2D for 12 months and concluded that this method can significantly increase the health promotion lifestyle score [31]. A similar study also exhibited that telenursing improved different domains of QOL related to endurance and physical health [47]. Moreover, Navicharern et al. assessed patients' satisfaction regarding the received T2DM treatment and concluded that telenursing can significantly increase patients' satisfaction [70]. Despite the results of previously mentioned studies, Blackberry et al. in 2013 conducted a study on 473 diabetic patients and divided them into the intervention and control groups (Table 4). The intervention group received weekly telephone calls for 18 months. Upon follow-up, patients' QOL did not differ significantly with telenursing intervention [36]. In line with their study, Hansen et al. observed no significant QOL changes following telenursing in PWT2D [58]. These contradictory results regarding QOL across the mentioned studies might be because of different questionnaires, different sample sizes, different study populations, and different follow-up periods.

Telenursing provides health education and directly involves patients in treatment plans regarding the guides given by nurses to patients and their families. Conversely, telenursing offers opportunities to monitor, follow up, and collect data, making room for multidisciplinary services such as remote intervention, pain management, and family support. Patient education and monitoring through telenursing methods improve self-care behaviors and treatment adherence in PWT2D, resulting in better diabetes control [71]. As a result, the rate of diabetes-related complications decreases, which leads to an increased QOL.

## 4. Conclusion

In conclusion, telenursing can be an effective supplement to the routine healthcare for the management of PWT2D. According to the literature, telenursing affects different areas of diabetes management including self-care, adherence to the treatment regimen, improvement of metabolic indicators, and quality of life. However, there is no consensus regarding the significant effect on body mass index and lipid profile. Telenursing is a direct communication method between the patients and healthcare staff who educate and monitor PWT2D. Increasing the knowledge of patients about their drugs, insulin administration, and diabetes complications improves self-care behaviors and medical adherence. Consistently, improved self-care and regular use of treatment result in improvement of metabolic indicators and decreased rate of complications, which is associated with better quality of life. Despite these promising results, the studies have utilized different methods. Further prospective controlled trials with larger number of patients are required to examine the efficacy of the strategies and confirm findings of the present review.

## Figures and Tables

**Table 1:** Main characteristics of studies assessing the impact of telenursing on self-care in type 2 diabetes patients.

Author, year country, ref.	Sex (M/F)	Age (mean)	Study design	Study population and setting	Follow-up time	Intervention group	Control group
Blackberry et al. (2013) (Australia) [36]	131/105	62.8	RCT	Patients with type 2DM, who were recruited at Patient Engagement And Coaching for Health study	12 weeks	Nurse telephone calls	Usual care
Elgaphar and El Gafar (2017) (Egypt) [31]	26/24	54	Quasiexperimental	Patients with type 2DM at least for 1 year, attending the endocrinology outpatient clinic and medical department of Menoufia University Hospitals	12 weeks	Routine care plus the telenursing program	Routine care
Shahabi et al. (2022) (Iran) [35]	14/16	46	RCT	Patients with type 2DM referring to diabetes clinic at Alzahra Hospital of Gilan Gharb.	8 weeks	Weekly nurse calls	Usual care
Azhdari Mamaghani et al. (2021) (Iran) [33]	—	55	RCT	Patients with type 2DM, attending the endocrinology outpatient clinic at Sina academic hospital of Tabriz	12 weeks	Nurse telephone calls and initial education	Usual care or initial nurse education without further telephone calls
Piette et al. (2000) (USA) [21]	48/76	56	Quasiexperimental	Patients with type 2DM with life expectancy longer than 1 year, attending the endocrinology outpatient clinic at Stanford University	12 months	Usual care plus biweekly automated assessment and self-care education	Usual care
Piette et al. (2001) (USA) [22]	95/31	60	RCT	Patients with type 2DM, attending the endocrinology outpatient clinic at Stanford University	12 months	Usual care plus biweekly assessment by nurse telephone calls	Usual care
Aliha et al. (2012) (Iran) [54]	16/15	50	RCT	Patients with type 2DM, attending the endocrinology outpatient clinic of Imam Khomeini Hospital	12 weeks	Nurse telephone calls	Routine care
Wong et al. (2005) (China) [23]	32/20	61	RCT	Patients with type 2DM, attending the endocrinology outpatient clinic at a regional hospital in Hong Kong	24 weeks	Routine care plus bi/weekly assessment by nurse telephone calls	Routine care
Aytekin et al. (2016) (Turkey) [34]	17/27	58	RCT	Patients with type 2DM, attending the endocrinology outpatient clinic at a regional hospital in Central Anatolia region	12 weeks	Education and nurse telephone follow-up	Routine care
Asante et al. (2020) (Ghana) [30]	8/22	55	RCT	Patients with type 2DM referring to the Diabetes Centre of Komfo Anokye Teaching Hospital in Kumasi	12 weeks	Phone follow-up calls by a diabetes specialist nurse	Self-Care education
Nesari et al. (2010) (Iran) [29]	19/11	51	RCT	Patients with type 2DM attending the Iranian Diabetes Society	12 weeks	Education and nurse telephone calls	Usual care
Soliman (2016) (Egypt) [32]	13/17	—	Quasiexperimental	Patients with type 2DM attending the outpatient endocrinology unit of medical hospital in Mansoura city.	12 weeks	Educational meetings by nurses and phone calls	Usual care

**Table 2:** Main characteristics of studies assessing the impact of telenursing on medication adherence in type 2 diabetes patients.

Author, year country, ref.	Sex (M/F)	Age (mean)	Study design	Study population and setting	Follow-up time	Intervention group	Control group
Crowley et al. (2013) (USA) [46]	56/126	56	RCT	African-American patients with type 2DM referring from the CHANGE study	12 months	Monthly nurse telephone calls	Usual care
Wong et al. (2005) (China) [23]	32/20	61	RCT	Patients with type 2DM, attending the endocrinology outpatient clinic at a regional hospital in Hong Kong	24 weeks	Routine care plus bi/weekly assessment by nurse telephone calls	Routine care
Aliha et al. (2012) (Iran) [54]	16/15	50	RCT	Patients with type 2DM, attending the endocrinology outpatient clinic of Imam Khomeini hospital	12 weeks	Nurse telephone calls	Routine care
Gatwood et al. (2016) (USA) [48]	12/12	47	RCT	Patients with type 2DM registered at a Western Michigan health system	12 weeks	Nurse telephone calls	Routine care
Soliman (2016) (Egypt) [32]	13/17	—	Quasiexperimental	Patients with type 2DM attending the outpatient endocrinology unit of medical hospital in Mansoura city.	12 weeks	Educational meetings by nurses and phone calls	Usual care
Asante et al. (2020) (Ghana) [30]	8/22	55	RCT	Patients with type 2DM referring to the Diabetes Centre of Komfo Anokye Teaching Hospital in Kumasi	12 weeks	Phone follow-up calls by a diabetes specialist nurse	Self-care education
Kaur et al. (2015) (India) [47]	21/19	51	RCT	Patients with type 2DM referring to the outpatient clinic	12 weeks	Nurse telephone calls	Routine care

**Table 3:** Main characteristics of studies assessing the impact of telenursing on metabolic indicators in type 2 diabetes patients.

Author, year country, ref.	Sex (M/F)	Age (mean)	Study design	Study population and setting	Follow-up time	Intervention group	Control group
Crowley et al. (2013) (USA) [46]	56/126	56	RCT	African-American patients with type 2DM referring from the CHANGE study	12 months	Monthly nurse telephone calls	Usual care
Stone et al. (2010) (USA) [57]	71/66	—	RCT	Patients with type 2DM, attending the endocrinology outpatient clinic at Sina Teaching Hospital of Tabriz	12 weeks	Nurse telephone calls	Usual care
Blackberry et al. (2013) (Australia) [36]	131/105	62.8	RCT	Patients with type 2DM, who were recruited at Patient Engagement And Coaching for Health study	12 weeks	Nurse telephone calls	Usual care
Esmaeilpour-BandBoni et al. (2020) (Iran) [51]	13/15	66	RCT	Patients with type 2DM admitted in an academic hospital in Rasht	12 weeks	Telenursing program	Routine care
Azhdari Mamaghani et al. (2021) (Iran) [33]	—	55	RCT	Patients with type 2DM, attending the endocrinology outpatient clinic at Sina academic hospital of Tabriz	12 weeks	Nurse telephone calls and initial education	Usual care or initial nurse education without further telephone calls
Lashkari et al. (2013) (Iran) [52]	—	55	Quasiexperimental	Patients with type 2DM, referring to the endocrinology outpatient clinics in Kerman	12 weeks	Nurse telephone calls	Usual care
Aliha et al. (2012) (Iran) [54]	16/15	50	RCT	Patients with type 2DM, attending the endocrinology outpatient clinic of Imam Khomeini hospital	12 weeks	Nurse telephone calls	Routine care
Elgaphar and El Gafar (2017) (Egypt) [31]	26/24	54	Quasiexperimental	Patients with type 2DM at least for 1 year, attending the endocrinology outpatient clinic and medical department of Menoufia University Hospitals	12 weeks	Nurse telephone calls	Routine care
Ravari et al. (2021) (Iran) [55]	—	64	RCT	Patients with type 2DM referring to Rafsanjan Diabetes Center	12 weeks	Nurse telephone calls	Routine care
Shahsavari and Foroghi (2020) (Iran) [50]	9/21	61	RCT	Illiterate patients with T2DM referring Aligoodarz clinic	12 weeks	Nurse telephone call	Routine care
Wong et al. (2005) (China) [23]	32/20	61	RCT	Patients with type 2DM, Attending the endocrinology outpatient clinic at a regional hospital in Hong Kong	24 weeks	Routine care plus/weekly assessment by nurse telephone calls	Routine care
Nesari et al. (2010) (Iran) [29]	19/11	51	RCT	Patients with type 2DM attending the Iranian Diabetes Society	12 weeks	Education and nurse telephone calls	Usual care
Asante et al. (2020) (Ghana) [30]	8/22	55	RCT	Patients with type 2DM referring to the Diabetes Centre of Komfo Anokye Teaching Hospital in Kumasi	12 weeks	Phone follow-up calls by a diabetes specialist nurse	Self-care education
Hansen et al. (2017) (Denmark) [58]	53/30	57	RCT	Patients with type 2DM referring to outpatient departments of three hospitals in Copenhaegen and the local health center	14 months	Monthly video calls with a healthcare nurse	Usual care
Kaur et al. (2015) (India) [47]	21/19	51	RCT	Patients with type 2DM referring to the outpatient clinic	12 weeks	Nurse telephone calls	Routine care
Navicharen (2009) (Thailand) [70]	20	NA	Quasiexperimental	Patients with type 2DM referring to two Red Cross stations in Bangkok	12 weeks	Nurse-based education and follow-up calls	Usual care
Odnoletkova et al. (2016) (Belgium) [59]	173/114	64	RCT	Patients with type 2DM affiliated with Belgian health insurance fund “Partena”	18 months	Nurse-based education and phone calls every 5 weeks	Usual care
Soliman (2016) (Egypt) [32]	13/17	—	Quasiexperimental	Patients with type 2DM attending the outpatient endocrinology unit of medical hospital in Mansoura city.	12 weeks	Educational meetings by nurses and phone calls	Usual care
Aytekin et al. (2016) (Turkey) [34]	17/27	58	RCT	Patients with type 2DM, attending the endocrinology outpatient clinic in a regional hospital in Central Anatolia region	12 weeks	Education and nurse telephone follow-up	Routine care

**Table 4:** Main characteristics of studies assessing the impact of telenursing on quality of life in type 2 diabetes patients.

Author, year country, ref.	Sex (M/F)	Age (mean)	Study design	Study population and setting	Follow-up time	Intervention group	Control group
Kaur et al. (2015) (india) [47]	21/19	51	RCT	Patients with type 2DM referring to the outpatient clinic	12 weeks	Nurse telephone calls	Routine care
Blackberry et al. (2013) (Australia) [36]	131/105	62.8	RCT	Patients with type 2DM, who were recruited at Patient Engagement And Coaching for Health study	12 weeks	Nurse telephone calls	Usual care
Hansen (2017) (Denmark) [58]	53/30	57	RCT	Patients with type 2DM referring to outpatient departments of three hospitals in Copenhagen and the local health center	14 months	Monthly video calls with a healthcare nurse	Usual care
Navicharen (2009) (Thailand) [70]	20	NA	Quasiexperimental	Patients with type 2DM referring to two Red Cross stations in Bangkok	12 weeks	Nurse-based education and follow-up calls	Usual care

## Data Availability

The data supporting this review are from previously reported studies and datasets, which have been cited. The data are available from the corresponding author upon request.
